# Phylogeographical patterns of a generalist acorn weevil: insight into the biogeographical history of broadleaved deciduous and evergreen forests

**DOI:** 10.1186/1471-2148-9-103

**Published:** 2009-05-16

**Authors:** Kyoko Aoki, Makoto Kato, Noriaki Murakami

**Affiliations:** 1 Graduate School of Human and Environmental Studies, Kyoto University, Sakyo-ku, Kyoto 606-8501, Japan; 2 Makino Herbarium, Tokyo Metropolitan University, Hachioji, Tokyo 192-0397, Japan

## Abstract

**Background:**

Climatic changes during glacial periods have had a major influence on the recent evolutionary history of living organisms, even in temperate forests on islands, where the land was not covered with ice sheets. We investigated the phylogeographical patterns of the weevil *Curculio sikkimensis *(Curculionidae), a generalist seed predator of Fagaceae plants living in both deciduous oak and evergreen forests of Japan. Its genetic structure was compared to that of another host-specific seed predator, *C. hilgendorfi*, inhabiting only evergreen forests.

**Results:**

We examined 921 bp of mitochondrial DNA for 115 individuals collected from 33 populations of *C. sikkimensis *from 11 plant species of three genera, *Quercus*, *Lithocarpus*, and *Castanopsis*. An analysis of molecular variance revealed that a large proportion (almost 50%, *P *< 0.001) of the total genetic variance could be explained by differences between two geographical regions, the southwestern and northeastern parts of the main islands of Japan. In contrast, no significant genetic differentiation of the weevil was observed among vegetation types of their utilized host plant species. The phylogeographical patterns of the generalist and the host-specific seed predator exhibited a congruent genetic boundary in the Chugoku-Shikoku region.

**Conclusion:**

Our results suggest that geology and historical environment have contributed to shaping the present genetic structure of *C. sikkimensis*. The geographical patterns of genetic differentiation in the Chugoku-Shikoku region observed in the two types of Fagaceae-associated *Curculio *in this study have also been observed in several plant species growing in warm and cool temperate zones of Japan. The occurrence of this common pattern suggests that deciduous oak and evergreen forests of Japan survived together, or adjacent to each other, in small refugia during glacial ages, in the southwestern and northeastern parts of the main islands, although these two types of forests are presently distributed in cool and warm temperate zones of Japan, respectively.

## Background

The current distribution of biological diversity cannot be understood without information about how organisms responded to historical geological and climatic conditions. In particular, the glacial-interglacial cycles of the Quaternary period may have played an important role in shaping the distribution of biodiversity among current populations, even in warm temperate zones, where the land was not covered with ice sheets [1,2]. The geological and geographical features of the Japanese Archipelago are comprised of several mountain ranges running largely parallel to a northeast to southwest axis. The mild coastal belt is close to these mountain ranges; thus the climate varies even in a narrow region. Consequently, a wide range of vegetation occurs, which accounts for various biogeographical factors that are not common on continental landmasses. Moreover, several landbridges between Japan and surrounding areas, which formed or disappeared in response to glacial-interglacial climatic changes, have also played important roles in determining the current distribution of Japanese biological diversity.

Phylogeographical patterns estimated from the extant genetic variation of organisms have proved highly informative in recovering the postglacial demographic histories of species [3,4]. Another powerful method is to compare intraspecific phylogeographical patterns among several taxa over the same area and to search for congruent geographic patterns of genetic variation, which would indicate the influence of common historical factors [5-17]. Comparing the intraspecific phylogeographical patterns among different species distributed in a single vegetation zone should be more informative because a group of species living together in the present environment likely responded in a similar or possibly the same manner to past geological or climatic events [5,18]. In the present study, we focused on the broadleaved deciduous and evergreen forest communities that characterize the biodiversity and endemism of the temperate zone, where the effects of climate change are particularly severe.

Palynological evidence indicates that broadleaved deciduous and evergreen forests in Japan were subjected to cold periods at least four times during the Quaternary [1,2]. During the glacial periods, climatic cooling caused southward and shifts to lower altitude in the geographic distribution of these forests. The pollen record indicates that refugial populations of broadleaved deciduous forests were sparsely distributed along the coasts of the Pacific Ocean and the Sea of Japan, while refugia of broadleaved evergreen forests were limited to southern areas mainly at the southern end of Kyushu. Fossil evidence also indicates that these populations migrated northward from refugia after the last glacial maximum [2,19,20]. These data suggest that populations of some forest-dependent insects, especially flightless or nonmigratory species, responded to the environmental changes associated with the glacial cycles in a similar fashion to that of their associated forests.

Most plant species growing in broadleaved deciduous and evergreen forests are parasitized by a diverse variety of herbivorous insects. We investigated and compared the mitochondrial DNA (mtDNA) genetic variation of two acorn weevils with different feeding types. These beetles are of particular interest because the females lay eggs in the host seeds. As a result, the fitness of the host and the weevil are intimately related [21]. The use of the rostrum during oviposition site preparation is considered the key adaptation that facilitates circumventing the physical defenses of the plant, such as the avoidance of attachment to the host [22]. All species of *Curculio *oviposit in seeds that are still on the tree. Females use the rostrum to excavate a hole in the seeds [23], after which they turn around and oviposit into the hole. The larvae feed on the seeds before leaving them to overwinter in the soil, where they pupate before emerging in spring or summer.

Five species of *Curculio *associated with Fagaceae seeds are distributed in Japan: *C. sikkimensis*, *C. hilgendorfi*, *C. dentipes*, *C. robustus*, and *C. conjugalis*. These include both generalist and host-specific seed predators of Fagaceae. Specifically, *C. sikkimensis *is a generalist associated with deciduous and evergreen trees [i.e., *Quercus *(deciduous and evergreen), *Lithocarpus *(evergreen), *Castanopsis *(evergreen), and *Castanea *(deciduous)], and *C. hilgendorfi *is a host-specific seed predator of *Castanopsis*. The other species are associated with several species of deciduous *Quercus*. *Curculio sikkimensis *and *C. hilgendorfi *are sometimes sympatrically distributed in evergreen forests.

In this study, we aimed to elucidate the biogeographical history of both types of forests by comparing the phylogeography of the two types (i.e., generalists and specialists) of acorn weevils. Our previous study showed that the mtDNA haplotypes found in the individuals of these two *Curculio *species, *C. sikkimensis *and *C. hilgendorfi*, validated each clade with high bootstrap support [24]. Moreover, our allozyme data showed that gene flow was disrupted between the two weevil species via reproductive isolation even in sympatric populations [24]. The two *Curculio *species exhibit similar adult morphologies, but can be identified by differences in the shape of the male genitalia [25]. Our previous study reported a phylogeographical and population demographic analysis of the host-specific seed predator *C. hilgendorfi *[24].

The purpose of this study was to assess the phylogeography of the generalist acorn weevil *Curculio sikkimensis *(HELLER) based on the current geographic distribution of mtDNA diversity. We report a phylogeographical pattern of *C. sikkimensis *collected from various Fagaceae seeds. This study addresses the following questions: 1] Do the mtDNA haplotypes of *C. sikkimensis *form host races? If *C. sikkimensis *exhibits some host specialization, the genetic differentiation of mtDNA in *C. sikkimensis *should be observable among their host Fagaceae species, among host plant genera, and between deciduous and evergreen host plants (i.e., deciduous and evergreen vegetation zones). 2] Do the phylogeographical patterns of *Curculio *species enable us to reconstruct the glacial and postglacial history of their associated temperate forests in Japan? Given that host plant species inhabit both broadleaved deciduous oak and evergreen forests, an investigation of the generalist predator *C. sikkimensis *provides an understanding of the biogeographical history of both deciduous oak and evergreen forests in Japan. However, the phylogeographical information of the specialist *C. hilgendorfi *reflects the history of evergreen forests. We compared the phylogeographical results of the generalist *C. sikkimensis *and the specialist *C. hilgendorfi *as well as those of the plant species growing to examine whether the evolutionary histories of their associated deciduous oak and evergreen forests are similar.

## Methods

### Insects

We sampled Fagaceae seeds of *Quercus*, *Lithocarpus*, and *Castanopsis *infested with *C. sikkimensis *from 33 localities in Japan (Table 1). Sampling was conducted during October and November 2002–2007, after larvae had grown up within the seeds. The seeds were kept in plastic bottles in the laboratory, where emerging larvae were collected. The larvae were preserved in 99% ethanol for DNA analysis.

**Table 1:** Sample collection sites and host plant species of *Curculio sikkimensis *in Japan

	Localities	Host plants	Latitude	Longitude	No. samples
1	Narita, Chiba	*Quercus acuta*	35.47.00	140.19.15	3
2	Mt. Takatsuka, Chiba	*Quercus acuta*	34.56.10	139.56.35	4
3	Higashizushi, Kanagawa	*Quercus acuta*	35.18.10	139.36.15	4
4	Uenohara, Yamanashi	*Quercus myrsinaefolia*	35.37.55	139.06.50	2
5	Ito, Shizuoka	*Castanopsis sieboldii *var.*sieboldii*	34.52.35	139.05.45	2
		*Quercus gilva*			1
6	Minamiizu, Shizuoka	*Castanopsis sieboldii *var.*sieboldii*	34.39.00	138.51.10	7
7	Minoshima, Wakayama	*Quercus variabilis*	34.04.40	135.07.25	1
8	Kitagawa, Kochi	*Quercus gilva*	33.28.50	134.03.25	2
9	Monobe, Kochi	*Quercus crispula*	33.48.15	133.53.40	6
10	Nangoku, Kochi	*Quercus gilva*	33.37.10	133.35.40	2
11	Kochi, Kochi	*Quercus glauca*	33.33.02	133.28.50	2
12	Susaki, Kochi	*Quercus glauca*	33.24.55	133.18.35	1
13	Himesaki, Sado Is., Niigata	*Castanopsis sieboldii *var.*sieboldii*	38.05.05	138.33.45	1
14	Futami, Sado Is., Niigata	*Castanopsis sieboldii *var.*sieboldii*	37.58.45	138.15.25	2
15	Hikone, Shiga	*Castanopsis cuspidata*	35.17.05	136.16.00	1
16	Mt. Hira, Shiga	*Quercus crispula*	35.12.50	135.53.35	5
17	Higashiyama, Kyoto	*Castanopsis cuspidata*	35.00.10	135.47.15	4
		*Quercus glauca*			1
18	Yoshida, Kyoto	*Quercus serrata*	35.01.35	135.47.05	4
		*Quercus glauca*			2
		*Castanopsis cuspidata*			1
19	Fushimi, Kyoto	*Castanopsis cuspidata*	34.58.05	135.46.55	1
20	Kasuga, Nara	*Quercus gilva*	34.40.40	135.51.25	4
		*Castanopsis cuspidata*			2
21	Oki Is., Shimane	*Quercus serrata*	36.10.35	133.18.10	4
22	Matsue, Shimane	*Quercus acuta*	35.28.55	133.03.05	2
23	Ooda, Shimane	*Quercus myrsinaefolia*	35.11.55	132.28.05	4
24	Kanayama, Okayama	*Castanopsis sieboldii *var.*sieboldii*	34.44.20	133.56.45	2
25	Kojima, Okayama	*Castanopsis cuspidata*	34.30.20	133.50.55	1
26	Niho, Yamaguchi	*Quercus gilva*	34.12.60	131.32.45	4
		*Castanopsis cuspidata*			2
27	Ogori, Yamaguchi	*Castanopsis cuspidata*	34.06.35	131.23.55	2
28	Kashii, Fukuoka	*Quercus gilva*	33.39.10	130.27.10	2
29	Kasuga, Fukuoka	*Quercus gilva*	33.31.20	130.28.10	3
30	Nobeoka, Miyazaki	*Quercus gilva*	32.37.15	131.34.35	3
31	Aya, Miyazaki	*Quercus salicina*	32.01.40	131.10.10	8
		*Quercus gilva*			5
		*Quercus acuta*			3
		*Lithocarpus edulis*			1
32	Issou, Yaku Is., Kagoshima	*Lithocarpus edulis*	30.27.15	130.29.10	3
33	Hanyama, Yaku Is., Kagoshima	*Quercus salicina*	30.22.10	130.23.05	1

### Sequencing of mtDNA

We analyzed sequence data for 115 *C. sikkimensis *individuals from 33 populations with 1–17 individuals per population (Table 1), from 11 plant species of the three genera, *Quercus*, *Lithocarpus*, and *Castanopsis*. Total DNA was extracted from the thoracic muscles of larvae using a standard proteinase K/SDS digestion and phenol-chloroform extraction. DNA fragments of the coding regions of mitochondrial cytochrome oxidase subunits I (COI) were amplified using the following PCR primers: COI-1729F, 5'-GGATCACCTGATATAGCATTCCC-3' and COI-2764R, 5'-CCTAAAAAATGTTGTGGGAAAAAGG-3' modified from [26]. Amplification of mtDNA was performed as follows: an initial 3 min denaturation (94°C) followed by 40 cycles of 0.5 min at 94°C, 0.5 min at 50°C, and 1 min at 70°C, and a final extension cycle of 10 min at 72°C. All PCRs were performed in a total volume of 20 μL using Ex-*Taq *polymerase (TaKaRa, Otsu, Japan), and the products were purified using ExoSAP-IT reagent (USB, Cleveland, OH, USA). Sequencing reactions were prepared using a BigDye terminator cycle sequencing kit (Applied Biosystems, Foster City, CA, USA) and analyzed on a PRISM 3100 sequencer (Applied Biosystems). Sequences [DDBJ accession nos. AB177451–AB177468, AB367602–AB367606, and AB457208–AB457233] were aligned using Sequence Navigator software (Applied Biosystems).

### Data analyses

Haplotype diversity (*h*), nucleotide diversity (π), and Tajima's *D *[27] were calculated using Arlequin version 2.0 [28]. A statistical parsimony network [29] was constructed to investigate relationships among haplotypes within *C. sikkimensis *using the program TCS[30]. Ambiguous loops in the network were resolved following the recommendations of [31]. An analysis of molecular variance (AMOVA) was used to examine the amount of genetic variability partitioned within and among populations as well as among groups of populations [32] using Arlequin. To assess the significance of the various factors that could affect partitioning of genetic variability, populations were grouped according to a set of grouping criteria, including geographic distribution, host plant genera, and vegetation types. The significance of fixation indices was tested using a nonparametric approach [32]. When AMOVA analysis could not determine what number of partitioned groups is the most suitable, a spatial analysis of molecular variance (SAMOVA) based on a simulated annealing procedure was used to define the groups of populations [33]. The program SAMOVA 1.0 iteratively seeks the composition of a user-defined number (*K*) of groups of geographically adjacent populations that maximizes Φ_CT_. The program was run for 10,000 iterations for *K *= 2 to 4 from each of 100 random initial conditions.

## Results

### Haplotype diversity and the geographical distribution of *C. sikkimensis*

**Figure 1:**
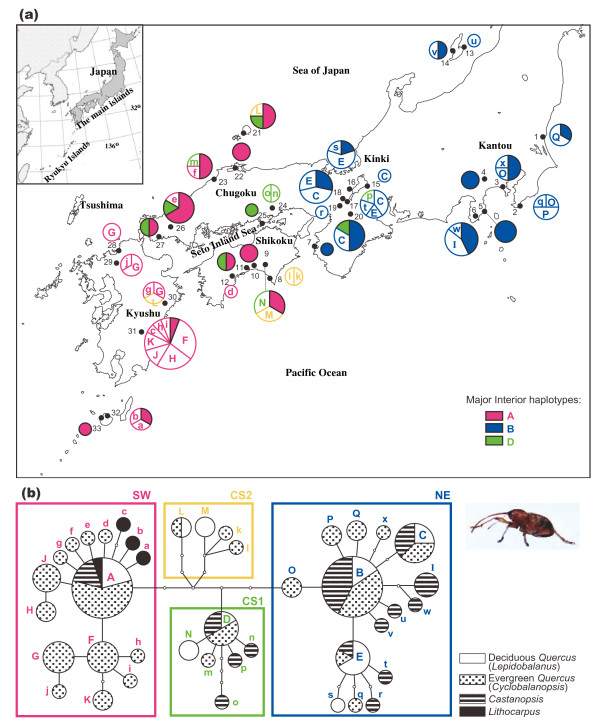
**Geographic distribution of mtDNA haplotypes of *Curculio sikkimensis***. (a) Geographic distribution of 41 mtDNA haplotypes found among 115 individuals of the generalist seed parasitic weevil, *C. sikkimensis*. Sample size ranged from 1 to 17 individuals per population. Circle sizes are proportional to haplotype frequencies. See Table 1 for the exact locations of populations and host plant species. (b) Relatedness among haplotypes detected in 921 bp of mtDNA (COI) of *C. sikkimensis*, represented in a statistical parsimony haplotype network. Each haplotype indicates the proportion of a host plant genus. Circle sizes are proportional to haplotype frequencies, and open dots indicate missing intermediate haplotypes.

We identified 47 variable sites in the mtDNA regions and 41 haplotypes in total, of which 24 haplotypes were found only once. The calculated values of haplotype diversity and nucleotide diversity are provided in Table 2. Haplotypes found in more than two individuals are denoted by uppercase letters, with the A-haplotype being the most common. Haplotypes found only once are denoted in lowercase letters. The geographical distribution of the recognized mtDNA haplotypes of *C. sikkimensis *was highly structured (Fig. 1a).

**Table 2:** Polymorphism summary statistics based on 921 bp of *Curculio sikkimensis *mtDNA

No. samples	No. haplotypes	Haplotype diversity (*h*)	Nucleotide diversity (π)	Tajima's *D*
115	41	0.9333	0.004654	-1.60560
				*P *= 0.024

The statistical parsimony haplotype network within *C. sikkimensis *with a 95% connection limit is shown in Fig 1b. Four main haplogroups (NE, CS1, CS2, and SW) were apparent in the network. Haplogroups NE and SW were found in the populations from northeastern (Kantou to Kinki district) and southwestern parts (Kyushu to the Chugoku-Shikoku region) of the main islands of Japan, respectively. Haplogroups CS1 and CS2 consisted of *C. sikkimensis *found in the Chugoku and Shikoku regions. The haplogroups NE, SW, and CS1 showed starlike shapes centered on haplotypes B, A, and D, respectively, which were the most frequently observed haplotypes.

### Genetic structure observed in *C. sikkimensis*

No significant genetic structure of *C. sikkimensis *was observed when compared to the genera of the host plant species. Most haplotypes were found from more than two individuals, and they were shared among the individuals of *C. sikkimensis *collected from different host plant genera of Fagaceae (Fig. 1b). For example, weevil individuals with haplotype A were found in the seeds of all types of host plant genera. Moreover, the AMOVA revealed no genetic differentiation by the vegetation types that included host plants (-0.03%, *P *> 0.10; Table 3) or among the functional host plant groups (6%, *P *> 0.10; Table 3). The results suggest that the differences in living vegetation zones as well as in utilized host plant species have not contributed to shaping the present genetic structure of *C. sikkimensis*.

**Table 3:** Analysis of molecular variance (AMOVA) of genetic variation in 921 bp of mtDNA of *Curculio sikkimensis*

Grouping criteria and source of variation	df	Sum of squares	Variance components	% of total variation	Φ statistics with *P*-value
**Geographic distribution I: [Kyushu, Chugoku to Shikoku] [Kinki to Kantou]**					
Among groups	1	91.7	1.56	52.8	Φ_CT _= 0.52
Among populations within groups	31	64.5	0.29	10.0	(*P *< 0.001)
Within populations	82	89.6	1.09	37.0	
Total	114	245.9	2.95		
					
**Geographic distribution II: [Kyushu] [Chugoku to Shikoku] [Kinki to Kantou]**					
Among groups	2	103.1	1.32	50.3	Φ_CT _= 0.50
Among populations within groups	30	53.2	0.21	8.1	(*P *< 0.001)
Within populations	82	89.6	1.09	41.5	
Total	114	245.9	2.62		
					
**Host plants: [Deciduous] [Evergreen]**					
Among groups	1	4.0	-0.07	-3.3	Φ_CT _= -0.03
Among populations within groups	38	157.6	1.09	51.0	(*P *> 0.10)
Within populations	75	84.2	1.12	52.3	
Total	114	245.9	2.14		
					
**Host plants: [Deciduous *Quercus*] [Evergreen *Quercus*] [*Castanopsis*] [*Lithocarpus*]**					
Among groups	3	25.5	0.15	6.8	Φ_CT _= 0.06
Among populations within groups	36	136.0	0.97	43.2	(*P *> 0.10)
Within populations	75	84.2	1.12	49.8	
Total	114	245.9	2.25		

Groupings of populations were examined based on their geographic distributions and host plants.

The geographical distributions of the recognized mtDNA haplotypes in *C. sikkimensis *were highly structured (Fig. 1a). The AMOVA showed strong genetic structure when grouping the populations according to their geographic distribution (Table 3). A large proportion (almost 50%, *P *< 0.001) of the total genetic variance was explained by differences among regions when comparing three geographical regions (i.e., Kyushu, locality numbers 28–33; Chugoku to Shikoku, locality numbers 21–27 and 8–12; and Kinki to Kantou, locality numbers 13–20 and 1–7) as well as two geographical regions (Kyushu to Chugoku-Shikoku, and Kinki to Kantou). The SAMOVA revealed that the value of Φ_CT _was larger at *K *= 2 than at *K *= 3, which indicated that partitioning into two groups was more appropriate. This result suggests that strong genetic differentiation exists between the southwestern and northeastern regions of the main islands of Japan.

### Comparison of the phylogeographical pattern in *C. sikkimensis *and *C. hilgendorfi*

**Figure 2:**
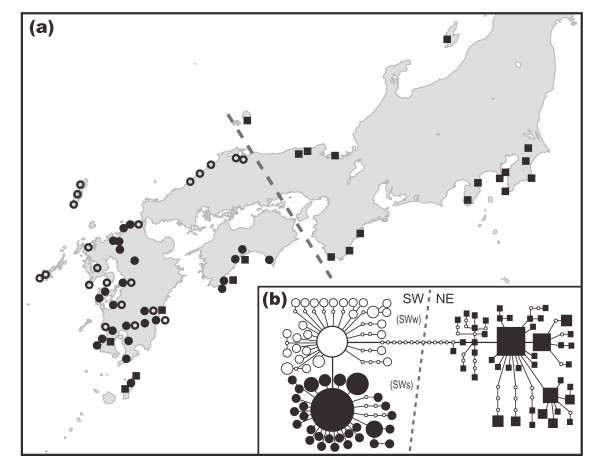
**Geographic distribution of mtDNA haplotypes of *Curculio hilgendorfi *on the main islands of Japan (modified from **[24]**)**. (a) Geographic distribution of 102 mtDNA haplotypes found among 189 individuals of the host-specific seed parasitic weevil *C. hilgendorfi *on the main islands of Japan. (b) Relatedness among haplotypes detected in 2709 bp of mtDNA (COI, COII, and ND5) of *C. hilgendorfi*, represented in a statistical parsimony haplotype network. The values of haplotype diversity and nucleotide diversity of NE and SW clades are 0.964 and 0.00385, respectively.

The haplotype and nucleotide diversities of the main islands of Japan showed similar values in *C. sikkimensis *(Table 2) and *C. hilgendorfi *(*h *= 0.964 and π = 0.00385, [24]). Congruent phylogeographical patterns were observed between *C. sikkimensis *(Fig. 1) and *C. hilgendorfi *(Fig. 2, [24]), with a deep gap between the SW and NE clades with respect to the mtDNA sequences. Two differences in the phylogeographical patterns were observed between *C. sikkimensis *and *C. hilgendorfi*. The first was the existence of CS clades in *C. sikkimensis *in the Chugoku and Shikoku regions, and the second was the lack of genetic differentiation in the SW clade of *C. sikkimensis *between the coasts of Pacific and the Sea of Japan.

## Discussion

### Phylogeographical patterns in *C. sikkimensis*

The observed gap between the SW and NE clades with respect to the mtDNA sequences of *C. sikkimensis *(Fig. 1) suggests that the southwestern and northeastern insect populations have been isolated for a long time, probably through several glacial and interglacial periods; the genetic groups have not been in contact for several thousands of years since the last glacial period; and long-distance migration of the weevil has been rare, resulting in negligible interpopulation genetic exchange events. This result suggests past fragmentation of this weevil species into at least two separate regions. Within the CS clade, the most common interior haplotype, D, was spread over the Chugoku and Shikoku regions. Genetic differentiation between the southwestern, northeastern, and Chugoku-Shikoku region was detected by the AMOVA. These results indicate the genetic uniqueness of the weevils distributed in the Chugoku-Shikoku region. Within the NE clade, a sub-haplogroup including the interior haplotype E was observed. These haplotypes also were often found in the Kinki region. Future studies examining more samples from this population may also reveal genetic uniqueness of the insects distributed in the Kinki region.

Molecular phylogenetic and ecomorphological studies on the genus *Curculio *in Europe and North America have suggested that the seed size of host plants is an important selective agent for changes in rostrum length and body size, and thus may be a key factor promoting morphological differentiation [21,34]. In our study, genetic differentiation of *C. sikkimensis *was not observed among the associated deciduous and evergreen forests or among host plant genera of Fagaceae (Table 3, Fig. 1b). These results suggest that genetic differentiation accompanied by host specialization among Fagaceae species is not occurring in *C. sikkimensis*, which is a uniquely polyphagous weevil species. Ecological and morphological studies of the weevils and their host plants in populations where various host plant species are sympatrically distributed may elucidate the evolution of Japanese acorn weevils.

### Phylogeographical pattern of the generalist and the host-specific seed parasitic weevils

A greater genetic differentiation between NE and SW clades was found for the specialist seed predator *C. hilgendorfi *compared to the generalist predator *C. sikkimensis *(Figs. 1b, 2). This was likely the result of the more limited geographical distribution of the host plants (*Castanopsis*) of the specialist *C. hilgendorfi*. A geographic boundary of the mtDNA haplotypes was observed in the Chugoku-Shikoku region in the two different host types of *Curculio*. Such boundaries were also observed in the chloroplast DNA of several plant species growing in warm and cool temperate zones in Japan [[5,35], T. Iwasaki et al. unpublished data]. In Europe, the Alps and Pyrenees may have blocked the dispersal of many animal and tree species from Italian and Iberian refugia to the northern part of Europe [reviewed in [10,17]]. However, in the case of the geographical boundary in the Chugoku-Shikoku region in Japan, neither a north-south mountain range nor a temperature gap has been observed. Other geographical and climatic features of the Seto Inland Sea, such as a currently drier climate and limited on-shore wind, and the historical existence of a grassland landscape during dry cool climates in the glacial ages, might have served as barriers against recolonization of various species from their refugia.

Two differences in phylogeographical patterns were observed between *C. sikkimensis *and *C. hilgendorfi*. *Castanopsis sieboldii *var.*sieboldii*, which is the major host plant species of *C. hilgendorfi*, is rarely distributed around the Seto Inland Sea. In the case of the host-specific seed predator *C. hilgendorfi*, the lack of distribution of the main host plant species around the Seto Inland Sea may have prompted genetic differentiation between *C. hilgendorfi *on the Pacific Coast and those on the coast of the Sea of Japan. In contrast, *C. sikkimensis *is a generalist seed predator of Fagaceae plants and could migrate across the Seto Inland Sea using various Fagaceae plant species in the Chugoku and Shikoku regions. This polyphagous habit allowed *C. sikkimensis *to cross the Seto Inland Sea and also may have generated the intermediate CS clades. A more detailed history of the area around the Seto Inland Sea can be estimated by comparing the phylogeographical patterns of various organisms with diverse geographic distributions and life histories.

### Insight into the glacial and postglacial history of temperate forests in Japan

Little phylogeographical data are available for the host plant species of these weevils due to the relatively slow rate of molecular evolution of plant chloroplast DNA [36-39], which is usually maternally inherited and thus often used in molecular phylogeographical studies. Extremely low levels of intraspecific variation in the chloroplast DNA of Japanese broadleaved evergreen species [40,41], including *Castanopsis *and *Lithocarpus*, as well as the possible introgression among *Quercus *species [41-43], have made it difficult to perform phylogeographical analyses of host plants. Among these host plant species, a phylogeographical pattern has been reported in deciduous *Quercus*; the geographic distribution of cpDNA haplotypes in deciduous *Quercus *indicated that one of the haplotypes is restricted to eastern Japan [44].

In this study *C. sikkimensis *living both in the broadleaved deciduous and evergreen forests and *C. hilgendorfi *living only in the broadleaved evergreen forests and in several plant species growing in these forests, shared a common phylogeographical pattern, with both exhibiting a genetic gap between the northeastern and southwestern parts of Japan. The geographic distributions of genetic diversity of plant species and those of their parasitic insects suggest that both were restricted to separate southwestern and northeastern refugia on the main islands of Japan during repeated glacial periods in the Quaternary. Moreover, these phytophagous insects, especially flightless or nonmigratory species, likely responded to the environmental changes associated with the glacial cycles in a fashion similar to that of their host plants. If the plant-insect association (i.e., the host range of the weevil) remained stable throughout the Quaternary, the presence of the phytophagous insects may be an indicator of the coverage of the temperate forests. Thus, incorporating phylogeographical information about herbivorous insect species with that of their host plant species could more precisely elucidate the phylogeographical patterns of their associated forests.

**Figure 3:**
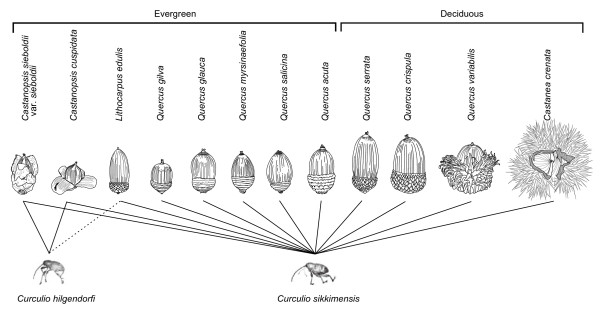
**Sizes and shapes of the acorns used by *Curculio sikkimensis *and *C. hilgendorfi***. The acorns and weevils have been drawn at an approximate scale of 1:2.

We did not find any genetic differentiation in the mtDNA of *C. sikkimensis *among their associated host plant species, which suggests that weevils occupying different host species do not shape host races. Although the size and shape of the utilized acorns varied among plant species (Fig. 3), *C. sikkimensis *can use almost all of these host plant species. Irrespective of the difference in host range between polyphagous *C. sikkimensis *and monophagous *C. hilgendorfi*, the phylogeographical patterns were very similar between the two species. This similarity is hypothesized to be due to (1) similar past distributions of refugia between deciduous oak and evergreen forests, or (2) similar past host ranges. That is, *C. sikkimensis *might have been restricted to evergreen *Castanopsis *species in the last glacial age and may have recently expanded its host range independently in each refugium after the last glacial maximum. In the former case, if some populations of *C. sikkimensis *survived during the glacial periods within deciduous oak forests and others in evergreen forests in different isolated refugia, some host specialization could have arisen or have been reinforced. The observation that the weevils inhabiting different acorn species do not shape host races would suggest that the populations of *C. sikkimensis *inhabiting deciduous oak forests and those in evergreen forests were not isolated during the long glacial periods. At the last glacial maximum, global sea level is estimated to have dropped to approximately 140 m lower than the present level [45]. The narrowing of the Tsushima Strait at that time caused a reduction in the Tsushima warm current into the Sea of Japan, resulting in further cooling and the aridification of the Japan Archipelago. Given this cool and arid climate, the areas covered by coniferous forests and cool-mixed forests expanded southwards [46]. This suggests that both deciduous oak and evergreen forests were restricted to the adjacent refugia at that time, although it is possible that the association between the insects and their host plant species could have established after the glacial period, as suggested by scenario 2 above. However, it is unlikely that *C. sikkimensis *expanded its host range to several deciduous and evergreen oak species independently from each refugium as recently as the last 20,000 years. In addition, in the European *Curculio *species, closely related weevils (e.g., *C. humeralis*, *C. pardalis*, and *C. victoriensis*) do not associate with common host plant species [21].

The coincident patterns of *C. sikkimensis *and *C. hilgendorfi *strongly support the hypothesis that both deciduous oak and evergreen forest types survived together or adjacent to one another in small refugia during glacial ages, although these forests are presently distributed in cool and warm temperate zones in Japan, respectively. A more detailed investigation of the pollen records or further genetic studies of organisms living in these types of forests will provide further insight into the evolutionary history of the broadleaved deciduous and evergreen forests of Japan.

## Conclusion

Our data suggest that geology and historical environment have contributed to shaping the recent genetic structure of *C. sikkimensis *in temperate forests of Japan. Comparing the phylogeographical patterns of two types of phytophagous insects (i.e., a generalist seed predator living in both the cool and warm temperate zones and the host-specific seed predator living only in the warm temperate zone) and of several plant species growing in these temperate zones of Japan enabled us to specify the historical processes of these forests more precisely. We demonstrated that during glacial ages the cool and warm temperate forests in Japan survived together, or adjacent to each other, at least in the southwestern and northeastern parts of the main islands. Our analyses provide a foundation for studying the evolutionary history of the broadleaved deciduous and evergreen forests in Japan, which characterize the biodiversity and endemism of the cool and warm temperate zones, where the effects of climate change are particularly severe.

## Authors' contributions

KA designed of the study, performed the molecular genetic studies, and drafted the manuscript. MK and NM helped in drafting the manuscript. All authors have read and approved the final manuscript.
